# Associations between Physical Activity Frequency in Leisure Time and Subjective Cognitive Limitations in Middle-Aged Spanish Adults: A Cross-Sectional Study

**DOI:** 10.3390/healthcare12111056

**Published:** 2024-05-22

**Authors:** Ángel Denche-Zamorano, Diana Salas-Gómez, Juan Manuel Franco-García, José Carmelo Adsuar, José A. Parraca, Daniel Collado-Mateo

**Affiliations:** 1 Promoting a Healthy Society Research Group (PHeSO), Faculty of Sport Sciences, University of Extremadura, 10003 Caceres, Spain; denchezamorano@unex.es (Á.D.-Z.);; 2 Departamento de Desporto e Saúde, Escola de Saúde e Desenvolvimento Humano, Universidade de Évora, 7004-516 Évora, Portugal; 3 Escuelas Universitarias Gimbernat (EUG), Physiotherapy School Cantabria, University of Cantabria, 39300 Torrelavega, Spain; 4 Health Economy Motricity and Education (HEME), Faculty of Sport Sciences, University of Extremadura, 10003 Caceres, Spain; 5 CIPER, Faculty of Human Kinetics, University of Lisbon, 1649-004 Lisbon, Portugal; 6 Comprehensive Health Research Centre (CHRC), University of Evora, 7004-516 Evora, Portugal; 7 Centre for Sport Studies, Rey Juan Carlos University, 28943 Madrid, Spain

**Keywords:** exercise, memory, cognition, dementia, prevention, physical activity

## Abstract

There is a global ageing of the world’s population. Ageing is associated with multiple pathologies, reductions in physical activity, and losses in cognitive function. This study aimed to analyse the associations between the frequency of leisure-time physical activity (PAF) in middle-aged Spaniards and subjective cognitive limitations (SCLs): self-reported problems for remembering or concentrating (data extracted from the 2017 National Health Survey and the 2020 European Health Survey in Spain). Furthermore, the study aimed to evaluate risk factors that could be related to a higher probability of developing SCLs. This was a cross-sectional study with 15,866 middle-aged Spaniards. The associations between FAP and SCLs were analysed using chi-square. Also, the risk factors for SCLs were evaluated using binary multiple logistic regression. The median age of participants was 55 years, with 49% men and 51% women. Associations were found between PAF and SCLs (*p* < 0.001). The highest prevalence of SCLs was found in physically inactive people and the lowest in very active people (13.7% vs. 5.8%, *p* < 0.05), and people with SCLs had a higher prevalence of inactivity than those without SCLs (47.2% vs. 33.8%, *p* < 0.05). Physical inactivity, low educational level, low social class, and being female were the main risk factors for SCLs. Among the actions to prevent cognitive limitations, as well as interventions in people with cognitive limitations, it would be advisable to include physical activity programmes, both as a preventive measure to delay cognitive limitations and to reduce the risk of other pathologies in people who already have them.

## 1. Introduction

According to the European Statistical Office (Eurostat), from 2012 to 2022, the average age of the European population increased by 2.5 years. In 2022, the median age of the EU’s population reached 44.4 years [1]. This increase in the average age of the population was higher in countries such as Portugal (+4.7 years), Spain (+4.3 years), Greece and Slovakia (+4.1 years), and Italy (+4.0 years) [1]. This problem is a global phenomenon and is increasing at an unprecedented rate in low- and middle-income countries [2]. Thus, the world’s population is ageing and in the coming years there will be more and more middle-aged and older adults.

### 1.1. Age and Cognitive Function

Ageing causes brain changes, e.g., in the prefrontal cortex and hippocampus, which affect cognitive abilities and cause cognitive impairment, e.g., losses in executive function and information processing, decreased attention span, reduced ability to concentrate, and problems with short- and long-term memory [3,4,5,6]. Although older age is associated with cognitive decline, longitudinal studies indicate that cognition begins to decline from early to middle age, after which the impairments in cognitive function are particularly pronounced, and gradually decline with age [7,8]. Changes in these cognitive functions contribute to a loss of quality of life and the ability to live independently [8].

It is therefore important to detect subjective cognitive limitations or impairments, which indicate how a person feels or experiences things, and assess changes in general cognitive function, such as memory, concentration, and others, in order to be able to offer early intervention [9]. Recent research suggests that subjective cognitive impairment is one of the important health problems to be managed from middle age onwards [9]. For example, the US Centers for Disease Control and Prevention (CDC) found that during 2015–2017, 10.8% of middle-aged people aged 45–64 years experienced subjective cognitive impairment [10].

### 1.2. Cognitive Function and Risk Factors

The evolution of cognitive alterations and the variability in these alterations between individuals depend on multiple factors and variables that can accelerate or decelerate processes of cognitive decline [11,12,13]. In addition to age itself, a number of risk factors have been identified that may accelerate cognitive decline [8]. It has been reported that women have a higher risk of dementia, Alzheimer’s, and cognitive impairment than men. Also, the progression from mild cognitive impairment to dementia is faster in women [14,15]. Another factor that has been associated with cognitive decline is educational level [16]. According to previous studies, it seems that a higher level of education appears to offer a protective effect against positive decline, probably because of a greater cognitive reserve than people with a lower level of education [8,16,17]. Other studies have found that higher socio-economic status may be a protective factor against cognitive decline [18,19,20,21,22]. Conversely, low socio-economic status appears to be a risk factor for cognitive impairment. These findings could be related to the smaller network of contacts, lower educational level, lower social support, or poorer nutrition that people with a low socio-economic level have, among other causes [18,19,20,21,22].

On the other hand, other research has presented obesity as a risk factor for cognitive impairment, although the available evidence is not sufficient to support this claim, as contradictory results have been found [23,24,25,26,27]. Relationships between marital status and cognitive function have also been studied, finding that being married may offer protection against lower cognitive function compared to being single, separated, or widowed [8,28,29,30,31], although environment and gender have an influence on these associations [30]. Lower cognitive impairment in married people may be related to greater social interactions [30]. Finally, disability [8], sleep disorders [32,33], depression [8], and hypertension [8] have also been reported to be associated with greater cognitive limitations.

### 1.3. Physical Activity and Cognitive Function

Physical inactivity has also been reported as an important risk factor for cognitive impairment [8]. Physical inactivity is defined as not engaging in sufficient physical activity within the current recommended guidelines (less than 150 min per week of moderate physical activity or less than 75 min per week of vigorous physical activity with energy expenditure greater than 3.0 metabolic equivalents (METs)) [34]. There is also clear evidence that physical inactivity is an independent risk factor for cardiovascular and metabolic diseases, mental illnesses, and other co-morbidities [35,36]. In turn, the presence of comorbidities can contribute to and has been found to be associated with cognitive decline and an accelerated loss of cognitive function in older people [37]. Reducing physical inactivity could prevent both cognitive decline and comorbidities that may contribute to or accelerate its onset [38].

On the other hand, physical activity (PA) is another modifiable lifestyle factor with potentially large beneficial effects on cognitive functioning. In addition, it appears to reduce the incidence of risk factors for dementia and has a positive impact on cognitive functioning [39,40]. It has been suggested that better functioning in many cognitive domains is associated with PA [39,40]. Despite these promising findings, the current evidence is inconsistent.

As previously mentioned, cognitive limitations are also present in middle-aged people. In this regard, several cross-sectional studies in middle-aged adults have shown that a higher level of PA is associated with higher scores in cognitive tasks [41,42,43]. However, there are studies that have not found an association between physical activity (moderate–vigorous) and cognitive function in the middle-aged population [44,45].

Given the controversial results found in the literature, a recent review of experimental studies suggests that caution should be exercised when relating the benefits of PA to cognition, as most of the available evidence comes from observational studies with a short follow-up period [46]. Another important factor in addition to intensity is the frequency of PA. In this sense, although less studied, it seems that compared to physical inactivity, performing physical activity once or several times a week is associated with better cognition [47].

The heterogeneity of the findings and the limited evidence available means that there is currently still a need for research into the relationship between cognitive function and PA in middle-aged people. Specifically, to the authors’ knowledge, there are no recent data assessing the relationship between cognitive function, taking into account the cognitive limitations that people experience, and the frequency of PA in the Spanish population.

Therefore, the present study aimed to assess the associations between the frequency of leisure-time physical activity (PAF) in middle-aged Spaniards and the presence of subjective cognitive limitations (SCLs) (self-reported problems for remembering or concentrating). In addition, the second objective was to assess the risk factors for having such experiences of subjective cognitive limitations. The hypotheses were that frequency of physical activity would be associated with cognitive limitations, and that physically inactive individuals would have a greater likelihood of experiencing cognitive limitations than the very active population.

## 2. Materials and Methods

### 2.1. Study Design

A secondary cross-sectional study was conducted based on primary published data from the 2017 Spanish National Health Survey (SNHS 2017) and the 2020 European Health Survey in Spain (EHSS 2020). STROBE recommendations were followed for the design and presentation of the study (Table S1). According to Regulation 2016/679 of the European Parliament and the Council of the European Union of 27 April 2016, on the protection of individuals concerning the processing of personal data and on the free movement of personal data, and derogating from Directive 95/46/EC, these data are public and anonymous and therefore considered as non-confidential data, and it was not necessary to apply data protection principles. The approval of an accredited ethics committee was not required.

### 2.2. Instruments (Spanish National Health Survey and European Health Survey in Spain)

The SNHS is a survey conducted by the Spanish Ministry of Health together with the National Statistical Institute (NSI), and the EHSS is conducted by the National Statistics Institute and coordinated by the European Statistical Office (Eurostat). The aim of both surveys is to determine the health status of the Spanish adult population. A stratified randomised sampling system was used in three phases. First, municipalities are grouped into strata, with the municipalities selected randomly. Subsequently, family dwellings are randomly selected from the selected municipalities. Finally, one adult is randomly selected from the selected family dwellings. All information and methodological details of the surveys are included in the methodologies of both surveys [48,49].

Participants who voluntarily agreed to participate were interviewed face to face by staff identified and trained by the NSI in completing the questionnaires. The surveys were conducted between October 2016 and October 2017 (SNHS 2017) and between July 2019 and July 2020 (EHSS 2020).

### 2.3. Sample

**Figure 1:**
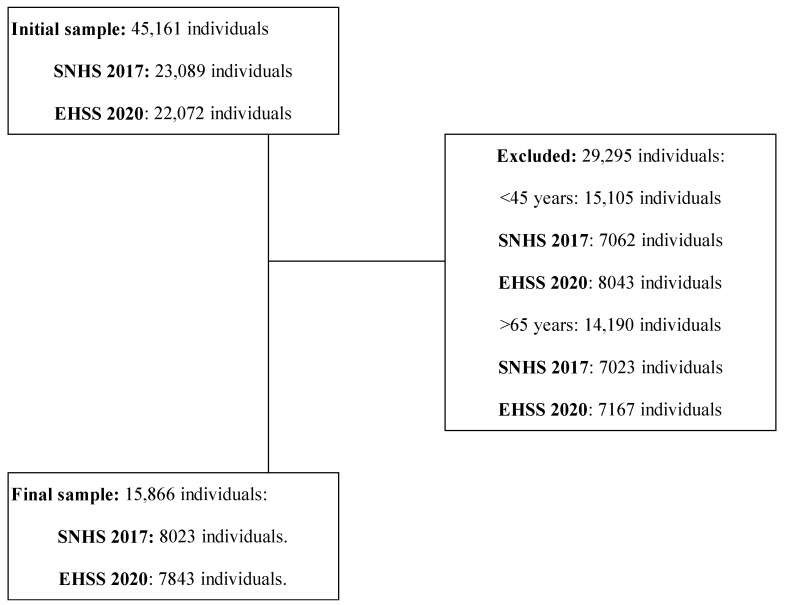
Flowchart showing the study sample’s eligibility criteria.

Data and responses to the questionnaires can be freely downloaded from the website of the Spanish NSI (SNHS 2017 and EHSS 2020). The SNHS 2017 and EHSS 2020 had final samples of 23,089 and 22,072 participants, respectively. All of them were adults aged 15 and over living in family dwellings in Spain. Once downloaded, the following inclusion criteria were applied to reach the final sample for the present study: (1) being a middle-aged adult (between 45 and 64 years old); (2) presenting data on cognitive limitations (response in item Q.38.a of both surveys). After applying these criteria, 29,295 persons were excluded (15,105 persons younger than 45 years and 14,190 persons older than 64 years), resulting in a final sample of 15,866 participants (8023 participants in SNHS 2017 and 7843 participants in EHSS 2020). Figure 1 shows the flow chart with the sample eligibility criteria.

### 2.4. Variables Extracted from the Surveys

#### 2.4.1. Outcome Variables

Subjective Cognitive Limitation Levels (SCLLs) were extracted from responses to the Q.38a variable: Do you have difficulty to remember or to concentrate? With 4 possible answers, as follows:
  (1) No, no difficulty (“None”).
  (2) Yes, some difficulty (“Some”).
  (3) Yes, many difficulties (“A lot”).
  (4) I can’t do it at all (“Absolutely”).

Or, Don’t know/don’t answer (DK/DA).

Subjective Cognitive Limitations (SCLs): This dichotomous variable was created from the responses to the SCLL variable. The results were grouped into 2 categories:
  (1) No: Participants who answered “No, no difficulty”.
  (2) Yes: Participants who answered “Yes, some difficulty”, or “Yes, many difficulties or can’t do it at all”.

#### 2.4.2. Independent, Predictor and Covariate Variables

Age: In years. This continuous variable was drawn from the variable “AGEa” of both surveys.
Sex: This was drawn from the variable “SEXOa” from both surveys with two possible responses (Men or Women).
Body Mass Index (BMI) Group: This was drawn from the variable “BMIa” from both surveys. Participants were grouped according to their BMI (Weight in kg/Height^2^ in metres). The following 4 groups were established:
  (1) Underweight (BMI < 18.5).
  (2) Normal (BMI ≥ 18.5 and <25).
  (3) Overweight (BMI ≥ 25 and <30).
  (4) Obesity (BMI ≥ 30).

Notably, 499 participants did not submit data on this variable.

Civil Status: This was drawn from the answers given by participants to item Q.4b: What is your legal marital status? There were 5 possible answers:
  (1) Single.
  (2) Married.
  (3) Widowed.
  (4) Legally Separated.
  (5) Divorced.

Or DK/DA. Fifty-five participants did not submit data on this variable.

Study Level: This was extracted from the variables “NIVEST” (SNHS 2017) and “STUDY” (EHSS 2020). These variables reflected the highest level of study attained by the participants. For this research, participants were grouped into 5 groups:
  (1) Primary studies (participants with completed or incomplete primary education).
  (2) Secondary studies (participants with compulsory secondary education with or without a diploma).
  (3) Baccalaureate (participants with baccalaureate studies).
  (4) Vocational training (participants with vocational education and training at intermediate or higher level or equivalent).
  (5) University (participants with university education).
Social Class: This was extracted from the variable “CLASE_PR” from both surveys. Both surveys grouped participants into six social classes (I, II, III, IV, V, and VI) according to their occupations as indicated. Table S2 shows a more comprehensive description of this classification.

Notably, 332 participants did not include data on this variable.

Physical Activity Frequency (PAF): This was taken from item Q.112 of both surveys. The question was as follows: Which of these possibilities best describes the frequency with which you do some physical activity in your free time? There were 4 possible answers. For this study, the groups were named as follows:
  (1) Never: Participants who answered “I do not exercise”.
  (2) Occasional: Participants who answered “I do occasional physical activity or sport”.
  (3) Frequently: Participants who responded “I do physical activity several times a month”.
  (4) Very frequently: Participants who responded “I do physical or sport training several times a week”.

Or DK/DA. Fourteen participants did not submit data on this variable.

Participants who did not submit data on any of these variables were excluded in the analyses that included the no data variable, although they were included in the rest of the analyses.

### 2.5. Statistical Analysis

The assumption of normality of the continuous variables was tested using the Kolmogorov–Smirnov test. Descriptive analysis of continuous and categorical variables was estimated using median (M) and interquartile range (IQR), and frequencies and proportions, respectively. These data are presented for the general population and by sex. The Mann–Whitney U-test was used to check differences in the age of men and women. The chi-squared test was used to analyse dependence relationships between sex and categorical variables and between PAF and subjective cognitive limitations (SCLs and SCLLs). In both cases, the post hoc pairwise z-test for independent proportions was performed to check differences in the proportions. PHI and Cramer’s V coefficients were calculated to interpret the strength of these associations.

To avoid or reduce confounding biases when analysing the odds of having subjective cognitive limitations, a multiple binary logistic regression was performed, taking the following as independent variables: Sex, Age, Civil Status, Study Level, BMI Group, and PAF. Subjective cognitive limitations was included as a dependent variable. Since the main objective was to evaluate the relationship between PAF (main exposure variable) and subjective cognitive limitations adjusted for several independent variables, we did not assess the interaction effects of these variables. *p* = 0.25 was the threshold for including variables in the multivariate model, as this has been suggested previously [50]. Their adjusted probability risks and 95% confidence intervals were calculated. For multiple logistic binary regressions, the assumptions of independence, no collinearity, and the absence of influential factors were tested. The model with the best goodness of fit according to the Hosmer–Lemeshow test was selected. IBM SPSS Statistics for Windows, Version 25.0. Armonk, NY, USA: IBM Corp. software was used and a value of *p* < 0.05 was considered significant.

## 3. Results

There was insufficient evidence to assume that the data for the age variable followed a normal distribution (*p* < 0.001). The sample had a median age of 55 years (IQR: 10), with no significant differences between men and women (*p* = 0.166). The sex of the participants was related to all sociodemographic variables: Civil Status (X^2^ = 349.3, df = 4, *p* < 0.001, V = 0.15), Study Level (X^2^ = 50. 7, df = 4, *p* < 0.001, V = 0.06), Social Class (X^2^ = 136.2, df = 5, *p* < 0.001, V = 0.09), and BMI Group (X^2^ = 812.0, df = 3, *p* < 0.001, V = 0.23) (Table 1).

Also, dependence relationships were found between sex and PAF (X^2^ = 50.2, df = 3, *p* < 0.001, V = 0.06). Men had higher proportions of PAF than women in the groups frequently (11.7% vs. 9.3%, *p* < 0.001) and very frequently (13.0% vs. 11.3%, *p* = 0.001). Dependence relationships were also found between sex and subjective cognitive limitations (X^2^ = 56.1, df = 1, *p* < 0.001, Φ = 0.06) and subjective cognitive limitation levels (X^2^ = 63.0, df = 3, *p* < 0.001, V = 0.06). Females had higher prevalences of subjective cognitive limitations (12.0% vs. 8.4%, *p* < 0.001) (Table 1).

### 3.1. Physical Activity Frequency and Subjective Cognitive Limitations

**Figure 2:**
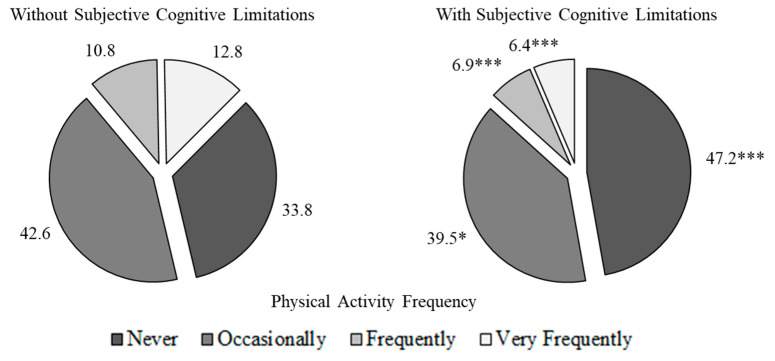
Physical activity frequency in relation to subjective cognitive limitations. *, *p*-value < 0.05; ***, *p*-value < 0.001.

PAF showed dependency relationships with subjective cognitive limitations (X^2^ = 146.5, df = 3, *p* < 0.001, V = 0.10) (Table S3). The group with SCLs showed higher proportions of inactive (never do exercise) people than the group without SCLs (47.2% vs. 33.8%, *p* < 0.001). In contrast, the proportion of people without cognitive limitations was higher in the very active group (12.8% vs. 6.4%, *p* < 0.001) (Figure 2).

**Figure 3:**
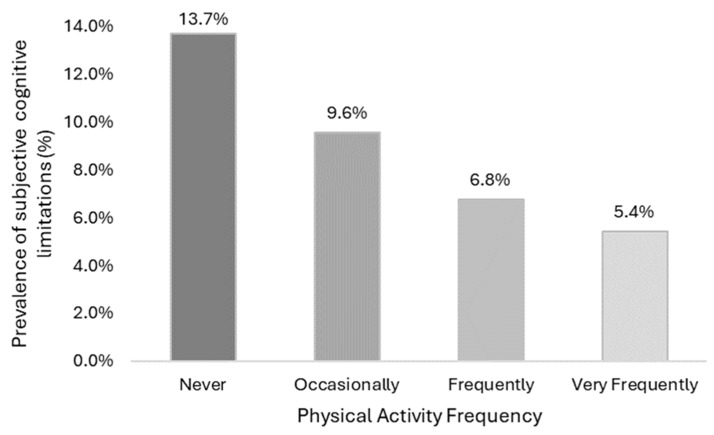
Prevalence of subjective cognitive limitations in relation to physical activity frequency.

As shown in Figure 3, the highest prevalence of subjective cognitive limitations was found in those who reported never doing PA compared to the group who did it occasionally (13.7% vs. 9.6%, *p* < 0.001) and the groups who did it frequently (6.8%, *p* < 0.001) and very frequently (5.4%, *p* < 0.001) (Table S4).

**Figure 4:**
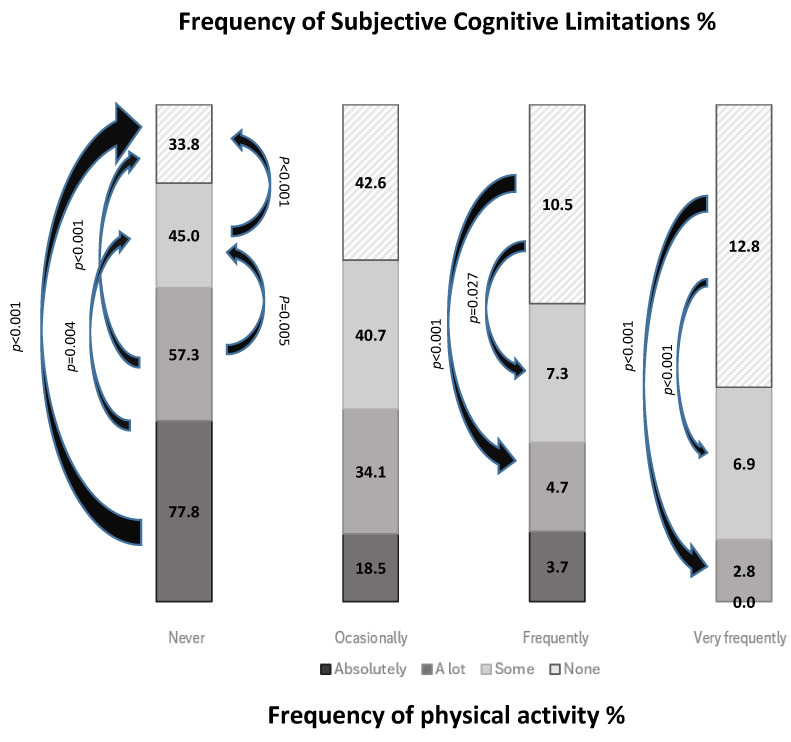
Physical activity frequency in relation to subjective cognitive limitation levels.

In addition, PAF was also found to be related to subjective cognitive limitation levels (X^2^ = 170.4, df = 9, *p* < 0.001, V = 0.06) (Table S5). Differences were found in the proportions of physical inactivity according to SCLLs (*p* < 0.001). Of the people who reported “Never” to performing PA, the proportion of them with absolutely SCLs (77.8%) was significantly higher than those who had some SCLs (45%, *p* = 0.004) and those who had no SCLs (33.8%, *p* < 0.001). Of those who reported “Frequently” in regard to PA, the proportion of people who reported having no SCLs (10.8%) was significantly higher than those who had some SCLs (7.3%, *p* < 0.001) and those who reported having a lot of SCLs (4.7%, *p* = 0.027). Of those who reported performing PA “Very frequently”, the proportion who reported having no SCLs (12.8%) was significantly higher than those who had some SCLs (6.9%, *p* < 0.001) and those who had a lot of SCLs (3.8%, *p* < 0.001) (Figure 4).

**Figure 5:**
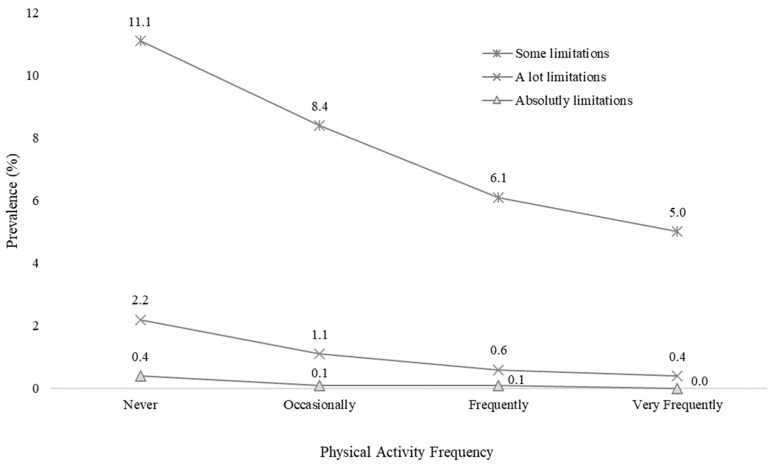
Subjective cognitive limitation levels in relation to physical activity frequency.

In addition, physically inactive people had higher prevalences of all levels of SCLs (Table S6), with the lowest prevalence found in the group that performed PA very frequently (Figure 5), followed by some SCLs (11.1% vs. 5.0%, *p* < 0.001), a lot (2.2% vs. 0.4%, *p* < 0.001), and absolutely (0.4% vs. 0.0%, *p* < 0.001).

### 3.2. Multiple Binary Logistic Regression

Table S7 shows the results of the multiple binary logistic regression. Age was significantly positively associated with subjective cognitive limitations (OR: 1.03; 95% CI: 1.02–1.04; *p* < 0.001). Furthermore, compared to men, women had a significantly higher probability of reporting subjective cognitive limitations (OR: 1.56; 95% CI: 1.39–1.74; *p* < 0.001). Compared to participants with a higher social class (I), those who belonged to social classes IV, V, and VI (1.44 ≤ OR ≤ 1.68, *p* < 0.001) had a higher probability of reporting subjective cognitive limitations. In addition, participants who never performed PA (OR: 2.09, 95% CI: 1.67–2.60, *p* < 0.001) and those who performed PA occasionally (OR: 1.49, 95% CI: 1.20–1.86, *p* < 0.001) had a higher probability of reporting subjective cognitive limitations than those who performed physical activity very frequently. Compared to married participants, single (OR: 1.46; 95%CI: 1.27–1.68; *p* < 0.001) participants had a higher probability of reporting subjective cognitive limitations.

**Figure 6:**
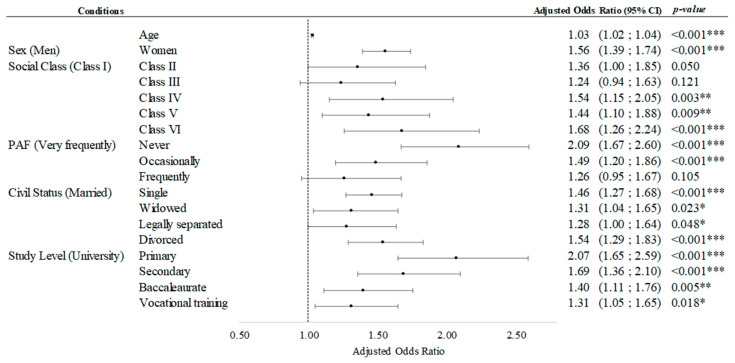
Multivariate binary logistic regression for subjective cognitive limitations. Adjusted Odds ratio (95 CI%); * *p*-value < 0.05; **, *p*-value < 0.01; ***, *p*-value < 0.001.

In terms of education level, those who did not have a university education had higher odds of having subjective cognitive limitations: primary (OR: 2.07, 95%CI: 1.65–2.59, *p* < 0.001), secondary (OR: 1.69, 95%CI: 1.36–2.10, *p* < 0.001), baccalaureate (OR: 1.40, 95%CI: 1.11–1.76, *p* = 0.005), and vocational training (OR: 1.31, 95%CI: 1.05–1.65, *p* = 0.018). Considering the odds ratios obtained in this model, the highest probabilities of reporting SCLs were found in the inactive group (OR: 2.09), those with primary education (OR: 2.07), those of lower class (OR: 1.68), women (OR: 1.56), divorcees (OR: 1.54), and older participants (OR: 1.03) (Figure 6).

## 4. Discussion

The present study aimed to assess the associations between subjective cognitive limitations (difficulty in remembering or concentrating) and the frequency of these limitations (some, a lot, absolute) with the frequency of leisure-time physical activity performed by middle-aged Spanish adults. Risk factors for having such experiences of subjective cognitive limitations were also assessed. Among the main results, a significant association was found between subjective cognitive limitations and the frequency of these limitations with PA.

### 4.1. Subjective Cognitive Limitations and Physical Activity in Leisure Time

Our results show that the highest proportions of subjective cognitive limitations were found in people who reported never doing physical activity in their leisure time. In contrast, the lowest proportions of reported cognitive limitations were found in those who reported doing physical activity “Very frequently” in their leisure time (I do physical or sport training several times a week). In line with our results, data are suggesting that self-reported PA is associated with better cognitive outcomes [46]. Previous cross-sectional studies have also reported that higher levels of physical activity are associated with higher scores in cognitive tasks [39,41,43]. Moreover, the study conducted by Xu et al. on 27,651 participants (50–85 years old) showed a dose–response relationship between PA and cognitive function [51]. After analysing the available evidence, it appears that the main cognitive skills that seem to be associated with PA are processing speed, memory, and executive function [39,41,43,52].

For example, the study by Spartano et al., using accelerometers to characterize physical activity levels, reports that PA improves cognitive performance in middle-aged people. Specifically, it appears that only 10–21.4 min of moderate-to-vigorous physical activity per day and total PA (which also includes activities of lower intensity) are associated with improved executive function in middle-aged people, but not visual memory or visual perception [41].

On the other hand, contrary to these and our results, a recent study by Quinlan et al. found that neither objectively measured nor self-reported physical activity (light, moderate, vigorous, or combined moderate-to-vigorous intensity) was associated with cognitive function. In this study, the cohort of subjects was relatively homogeneous in age, education, and socio-economic status (healthy middle-aged adults (40.6 years on average) with a good education) [53]. In contrast, in our population, the median age was older (54 years) and the population was heterogeneous in terms of educational level, level of studies, etc., making it more representative with a greater capacity for extrapolation to the general middle-aged population. Differences in sample characteristics and the tools used to quantify PA may explain the differences in results.

Following these findings, some authors suggest that physical activity in midlife may be most predictive of future cognitive performance, but that the cognitive tasks used are not sensitive enough to detect more subtle effects that take place in midlife [53]. One of the difficulties in assessing healthy middle-aged adults with neurocognitive tests is that ceiling effects tend to be frequent in this population [54]. Because of this, self-reported cognitive limitations seem to be an appropriate way to detect cognitive impairments in this population [9].

In relation to these studies, it has also been discussed in the literature which is the best way to quantify PA (objective versus subjective), with the conclusion that both methods may be used to assess different aspects of PA [55]. In particular, objective measures, with accelerometers being most commonly used, seem to be unable to detect some types of activities due to their location on the body, e.g., upper body movements when worn at the waist. In contrast, subjective measures can lead to a more comprehensive description of weekly PA in terms of type of activity, time frame during the daily and weekly routine, and self-perception of involvement in physical activity [55]. However, they are limited in their ability to measure PA intensity.

It should be noted that, in this study, although the associations between PA and subjective cognitive limitations were significant, the strength of these associations was low. Similar results in a recent meta-analysis including prospective cohort and case–control studies found that the association between physical activity and cognitive decline was very small, with no clear dose–response association in middle-aged people [56]. However, even weak associations can be clinically significant from a health perspective. Therefore, despite the inconsistency of the current results, the authors consider it relevant and necessary to promote strategies to improve the modifiable risk factor of physical inactivity for cognitive decline in middle-aged people.

At present, comparison between studies is challenging and the interpretation of results is complex. These difficulties lie mainly in the different methodologies used in studies assessing the association between cognitive function and PA, for example, differences in the tools used to quantify PA (objective vs. subjective), as well as great heterogeneity in the samples analysed, including variations in age range, education, socio-economic status, and activity levels. Therefore, it is necessary to take these possible factors into account when designing preventive strategies against cognitive impairment through PA. Preventive strategies should be adapted to the socio-demographic and socio-economic differences of each individual.

#### Logistic Regression Analysis

After adjusting the analyses for other factors in addition to PAF, such as age, sex, social class, marital status, and educational level, being physically inactive was the significant risk factor with the highest probability (OR: 2.09) of presenting subjective cognitive limitations (difficulty in remembering and concentrating).

Similar results, although the methodology used to quantify PA was different (objective by accelerometry), have been reported by Vásquez et al. Although they found no association between higher levels of PA and cognition, they did observe a significant association between cognitive function and sedentary behaviours. As in the present study, the association was more evident in sedentary middle-aged women than in men [45]. Also in line with our results, an observational study of 93,082 respondents aged ≥45 years in the US reported that people with self-reported cognitive impairment were more frequently inactive than those without this subjective cognitive impairment status [44].

In addition, in the present study, other factors such as a low level of education, belonging to a lower social class, being divorced, being female, and being older were potential factors that increased the probability of reporting cognitive limitations. In part, the results of this study support previous studies that reported an increased risk of subjective cognitive impairment in female participants and those with older age, lower education level, and lower income [9,57].

In this study, those who reported a primary education level were 2.07 times more likely to have subjective cognitive limitations. The study by Lin et al. reports that females with primary or lower education levels are closely linked to subjective cognition decline symptoms. A hypothesis is that people with a good educational level have a higher brain capacity than people with a lower educational level, which gives them the advantage of slowing cognitive decline [22]. On the other hand, the fact that women are more likely to present a higher probability has also been related to the fact that women are more concerned about their state of health and have a greater perception of changes or the progression of symptoms [58]. They may therefore be more likely to self-report having cognitive difficulties.

Similar results were also observed in a study by Liu et al., in which elderly people with widowed/separated marital status were more susceptible to cognitive impairment and performed poorly in all domains of cognition [59]. On the other hand, the study by Shi et al. also reported that older people who had a disadvantaged socio-economic status were more likely to develop a lower level of cognitive ability in later life after controlling the results for age, sex, marital status, living region, health insurance, lifestyle factors, and physical health status [22].

### 4.2. Limitations and Future Lines of Research

There is great potential in our results as a result of the robustness of the results and the representativeness of the sample analysed. However, this study had some limitations. As a cross-sectional study, we were unable to establish causal relationships between frequency of physical activity and cognitive limitations. In both the SNHS and the EHSS, cognitive limitations and frequency of physical activity were obtained through questionnaires, and it was the participants who reported their cognitive limitations and frequency of physical activity. It would be advisable for these surveys to also include tests that assess participants’ cognitive functions, in addition to being able to record objective data on participants’ levels of physical activity. It would be interesting to have both objective and subjective data to characterize both variables in this population. In addition, on 17 March 2020, confinement in Spain was established. From that moment on, the interviews were changed from face-to-face to telephone interviews, which could have affected the results. The limitations of this study create opportunities for future research. Based on the results of this study and taking into account the limitations mentioned above, the availability of these data could help to promote community health strategies and implement prevention programmes aimed at reducing physical inactivity and inactive behaviours in order to improve the cognitive health of the Spanish middle-aged population. The authors consider that being able to maintain an optimal frequency of physical activity during this period of life could play a protective role against age-related cognitive decline. In addition, it is necessary to know and take into account the factors that affect subjective cognitive limitations and decline in middle-aged adults in order to improve approaches to prevent cognitive decline and dementia.

## 5. Conclusions

Cognitive decline in middle-aged people is related to multiple factors, including physical activity. More than 75% of the Spanish middle-aged population report undertaking physical activity in their leisure time occasionally (35%) or never (42%).

Physical inactivity is associated with losses in cognitive function. This study found dependence relationships between cognitive limitations and frequency of physical activity. The prevalence of cognitive limitations in inactive people (13.7%) was more than twice as high as in those who engaged in leisure-time physical activity frequently (6.8%) or very frequently (5.4%).

Risk factors associated with cognitive limitations included physical inactivity, low educational level, low social class, being single or divorced, being female, and age. Cognitive impairment prevention and intervention programs should include physical activity, social support, and cognitive stimulation, among other enabling elements.

## Figures and Tables

**Table 1:** Descriptive analysis of the whole sample by sex for middle-aged Spanish men and women.

Variables	Total = 15,866		Men = 7765		Women = 8101		X^2^	df	*p*	V
	Median	IQR	Median	IQR	Median	IQR				
Age (Years)	55	(10)	54	(10)	55	(10)	n.a.	n.a.	0.166	n.a.
**Civil Status**	**n**	**%**	**n**	**%**	**N**	**%**	**X^2^**	**df**	*p* *	**V**
Single	2883	18.2	1628	21.0	1255	15.6 ***	349.3	4	<0.001	0.15
Married	10,074	63.7	5035	65.0	5039	62.5 **				
Widowed	692	4.4	127	1.6	565	7.0 ***				
Legally separated	678	4.3	306	4.0	372	4.6 *				
Divorced	1484	9.4	648	8.4	836	10.4 ***				
**Study Level**										
Primary	3149	19.8	1538	19.8	1611	19.9	50.7	4	<0.001	0.06
Secondary	4396	27.7	2288	29.5	2108	26.0 ***				
Baccalaureate	2175	13.7	1073	13.8	1102	13.6				
Vocational training	2768	17.4	1379	17.8	1389	17.1				
University	3378	21.3	1487	19.2	1891	23.3 ***				
**Social Class**										
I	1788	11.5	855	11.1	933	11.9	136.2	5	<0.001	0.09
II	1259	8.1	590	7.7	669	8.5				
III	3155	20.3	1436	18.7	1719	21.9 ***				
IV	2070	13.3	1213	15.8	857	10.9 ***				
V	5056	32.5	2616	34.1	2440	31.0 ***				
VI	2206	14.2	959	12.5	1247	15.9 ***				
**BMI Group**										
Underweight	191	1.2	33	0.4	158	2.0 ***	812.0	3	<0.001	0.23
Normal	5925	38.6	2145	28.3	3780	48.6 ***				
Overweight	6389	41.6	3817	50.3	2572	33.1 ***				
Obesity	2862	18.6	1597	21.0	1265	16.3 ***				
**PAF**										
Never	5579	35.2	2740	35.3	2839	35.1	50.2	3	<0.001	0.06
Occasionally	6696	42.2	3102	40.0	3594	44.4 **				
Frequently	1655	10.4	905	11.7	750	9.3 ***				
Very frequently	1922	12.1	1010	13.0	912	11.3 **				
**Subjective Cognitive Limitations Level**										
No	14,246	89.8	7115	91.6	7131	88.0 ***	63.0	3	<0.001	0.06
Yes, something	1382	8.7	537	6.9	845	10.4 ***				
Yes, a lot	211	1.3	98	1.3	113	1.4				
Yes, absolutely	27	0.2	15	0.2	12	0.1				
**Subjective Cognitive Limitations**							**X^2^**	**df**	***p***	**Φ**
No	14,246	89.8	7115	91.6	7131	88.0 *	56.1	1	<0.001	0.06
Yes	1620	10.2	650	8.4	970	12.0 ***				

IQR, interquartile range; n, participants; %, percentage; PAF, physical activity frequency; *p*, *p*-value from Mann–Whitney U test; *p* *, *p*-value from chi-square test; *, significant differences of proportions between sex from post hoc pairwise z-test for independent proportions with *p* < 0.05; **, *p*-value < 0.01; ***, *p*-value < 0.001; X^2^, chi-square statistic; df, degree of freedom; V, Cramer’s V coefficient; Φ: Phi coefficients; n.a., not application.

## Data Availability

Data will be made available upon reasonable request to the corresponding author.

## Supplementary Materials

The following supporting information can be downloaded at: https://www.mdpi.com/article/10.3390/healthcare12111056/s1, Table S1. STROBE Statement—Checklist of items that should be included in reports of cross-sectional studies; Table S2. Description of social classes based on occupational occupation; Table S3. Prevalence of physical activity frequency according to Subjective Cognitive Limitations; Table S4. Prevalence of memory problems according to physical activity frequency; Table S5. Subjective Cognitive Limitations Levels according to Physical Activity Frequency; Table S6. Subjective Cognitive Limitations Levels according to Physical Activity Frequency; Table S7. Multivariate binary logistic regression analysis including Subjective Cognitive limitations as the dependent variable.
