# Supplementary material for: Associations between Physical Activity Frequency in Leisure Time and Subjective Cognitive Limitations in Middle-Aged Spanish Adults: A Cross-Sectional Study
Source: Healthcare (Basel). 2024 May 22;12(11):1056. doi: 10.3390/healthcare12111056 (PMC11171578; doi:10.3390/healthcare12111056)
Supplement: Supplementary file 1 [file healthcare-12-01056-s001.zip › Table S2. Description of social classes based on occupational occupation.pdf]

Table S2. Description of social classes based on occupational occupation.

**CLASS I** - Directors and managers of establishments with 10 or more employees, and professionals traditionally associated with university degrees
**CLASS II** - Directors and managers of establishments with fewer than 10 employees, professionals traditionally associated with university degrees and other technical support professionals. technical support professionals. Athletes and artists
**CLASS III** - Intermediate occupations and self-employed workers
**CLASS IV** - Supervisors and workers in skilled technical occupations
**CLASS V** - Skilled workers in the primary sector and other semi-skilled workers semi-skilled workers
**CLASS VI** - Unskilled workers
