# Supplementary material for: Associations between Physical Activity Frequency in Leisure Time and Subjective Cognitive Limitations in Middle-Aged Spanish Adults: A Cross-Sectional Study
Source: Healthcare (Basel). 2024 May 22;12(11):1056. doi: 10.3390/healthcare12111056 (PMC11171578; doi:10.3390/healthcare12111056)
Supplement: Supplementary file 1 [file healthcare-12-01056-s001.zip › Table S3. Prevalence of physical activity frequency according to Subjective Cognitive Limitations.pdf]

Table S3. Prevalence of physical activity frequency according to Subjective Cognitive Limitations

Variables	Subjective Cognitive Limitations				X^2	df	p	V
PAF	NO SCL (A)		SCL (B)					
	n	%	n	%				
Never	4,815	33.80%	764	47.20%	146.5	3	<0.001	0.10
Occasionally	6,056	42.60%	640	39.50%				
Frequently	1,543	10.80%	112	6.90%				
Very Frequently	764	12.80%	640	6.40%				
Proportions' differences post hoc								
Never			A (p<0.001)***					
Occasionally	B (p=0.019)*							
Frequently	B (p<0.001)***							
Very Frequently	B (p<0.001)***							

p (p-value from pairwise z-test for independent proportions); * (p<0.05); *** (p<0.001); X2 (Chi-Square); df (Degree freedom); V (V's Cramer coefficients).
