# Supplementary material for: Associations between Physical Activity Frequency in Leisure Time and Subjective Cognitive Limitations in Middle-Aged Spanish Adults: A Cross-Sectional Study
Source: Healthcare (Basel). 2024 May 22;12(11):1056. doi: 10.3390/healthcare12111056 (PMC11171578; doi:10.3390/healthcare12111056)
Supplement: Supplementary file 1 [file healthcare-12-01056-s001.zip › Table S4. Prevalence of memory problems according to physical activity frequency.pdf]

Table S4. Prevalence of subjective cognitive limitations according to physical activity frequency.

Variables	PAF								X^2	df	p	V
Subjective cognitive limitations	Never (A)		Occasionally (B)		Frequently (C)		Very Frequently (D)					
	n	%	n	%	n	%	n	%	146.5	3	<0.001	0.096
YES	764	13.70%	640	9.60%	112	6.80%	104	5.40%				
Proportions' differences post hoc												
Proportions' differences		B (p<0.001)***		C (p=0.002)**								
		C (p<0.001)***		D (p<0.001)***								
		D (<0.001)***										

p (p-value from pairwise z-test for independent proportions); ** (p<0.01); *** (p<0.001); X^2 (Chi-Square); df (Degree freedom); V (V's Cramer coefficients).
