# Supplementary material for: Associations between Physical Activity Frequency in Leisure Time and Subjective Cognitive Limitations in Middle-Aged Spanish Adults: A Cross-Sectional Study
Source: Healthcare (Basel). 2024 May 22;12(11):1056. doi: 10.3390/healthcare12111056 (PMC11171578; doi:10.3390/healthcare12111056)
Supplement: Supplementary file 1 [file healthcare-12-01056-s001.zip › Table S5. Subjective Cognitive Limitations Levels according to Physical Activity Frequency (2).pdf]

Table S5. Subjective Cognitive Limitations Levels according to Physical Activity Frequency.

| Variables | Subjective Cognitive Limitations Levels | | | | | | | | $X^2$ | df | p | V |
|---|---|---|---|---|---|---|---|---|---|---|---|---|
| Physical Activity Frequency | None (A) | | Some (B) | | A lot (C) | | Absolutely (D) | | | | | |
| | n | % | n | % | n | % | n | % | | | | |
| Never | 4,815 | 33.8% | 622 | 45.0% | 121 | 57.3% | 21 | 77.8% | 170.4 | 9 | <0.001 | 0.06 |
| Occasionally | 6,056 | 42.6% | 563 | 40.7% | 72 | 34.1% | 5 | 18.5% | | | | |
| Frequently | 1,543 | 10.8% | 101 | 7.3% | 10 | 4.7% | 1 | 3.7% | | | | |
| Very Frequently | 1,818 | 12.8% | 96 | 6.9% | 8 | 3.8% | 0 | 0.0% | | | | |
| Proportions' differences post hoc | | | | | | | | | | | | |
| Never | | | A (p<0.001) *** | | A (p<0.001) ***<br>B (p=0.005) ** | | A (p<0.001) ***<br>B (p<0.004) ** | | | | | |
| Occasionally | B (p<0.001) ***<br>C (p<0.027) * | | | | | | | | | | | |
| Frequently | B (p<0.001) ***<br>C (p<0.001) *** | | | | | | | | | | | |
| Very Frequently | B (p=0.001) ** | | | | | | | | | | | |

p (p-value from pairwise z-test for independent proportions); * (p(0.05)** (p<0.01); *** (p<0.001); $X^2$ (Chi-Square); df (Degree freedom); V (V's Cramer coefficients).
