# Supplementary material for: Associations between Physical Activity Frequency in Leisure Time and Subjective Cognitive Limitations in Middle-Aged Spanish Adults: A Cross-Sectional Study
Source: Healthcare (Basel). 2024 May 22;12(11):1056. doi: 10.3390/healthcare12111056 (PMC11171578; doi:10.3390/healthcare12111056)
Supplement: Supplementary file 1 [file healthcare-12-01056-s001.zip › Table S6. Subjective Cognitive Limitations Levels according to Physical Activity Frequency..pdf]

Table S6. Subjective Cognitive Limitations Levels according to Physical Activity Frequency.

Variables	PAF								X^2	df	p	V
Subjective Cognitive Limitations Levels	Never (A)		Occasionally (B)		Frequently (C)		Very Frequently (D)					
	n	%	n	%	n	%	n	%				
None	4,815	86.3%	6,056	90.4%	1,543	93.2%	1,818	94.6%	170.4	9	<0.001	0.06
Some	622	11.1%	563	8.4%	101	6.1%	96	5.0%				
A lot	121	2.2%	72	1.1%	10	0.6%	8	0.4%				
Absolutely	21	0.4%	5	0.1%	1	0.1%	0	0.0%				
Proportions' differences post hoc												
None				A (p<0.001) ***		A (p<0.001) ***<br>B (p=0.002) **		A (p<0.001) ***<br>B (p<0.001) ***				
Some		B (p<0.001) ***<br>C (p<0.001) ***<br>D (p<0.001) ***		C (p=0.011) *<br>(p<0.001) ***	D							
A lot		B (p<0.001) ***<br>C (p<0.001) ***<br>(p<0.001) ***	D	D (p=0.047) *								
Absolutely		B (p=0.001) **										

p (p-value from pairwise z-test for independent proportions); * (p<0.05); ** (p<0.01); *** (p<0.001); X^2 (Chi-Square); df (Degree freedom); V (V's Cramer coefficients).
