# Supplementary material for: Associations between Physical Activity Frequency in Leisure Time and Subjective Cognitive Limitations in Middle-Aged Spanish Adults: A Cross-Sectional Study
Source: Healthcare (Basel). 2024 May 22;12(11):1056. doi: 10.3390/healthcare12111056 (PMC11171578; doi:10.3390/healthcare12111056)
Supplement: Supplementary file 1 [file healthcare-12-01056-s001.zip › Table S7. Multivariate binary logistic regression analysis including Subjective Cognitive limitations as the dependent variable.pdf]

Table S7. Multivariate binary logistic regression analysis including Subjective Cognitive limitations as the dependent variable.

	β	Adjusted OR	(95% CI)	p
Age	0.031	1.03***	(1.02; 1.04)	<0.001
Sex (Men)		Ref.		
Women	0.443	1.56***	(1.39; 1.74)	<0.001
Social Class (I)		Ref.		
II	0.306	1.36	(1.00; 1.85)	0.050
III	0.215	1.24	(0.94; 1.63)	0.121
IV	0.432	1.54**	(1.15; 2.05)	0.003
V	0.363	1.44**	(1.10; 1.88)	0.009
VI	0.521	1.68***	(1.26; 2.24)	<0.001
PAF (Very frequently)		Ref.		<0.001
Never	0.735	2.09***	(1.67; 2.60)	<0.001
Occasionally	0.400	1.49***	(1.20; 1.86)	<0.001
Frequently	0.232	1.26	(0.95; 1.67)	0.105
Civil Status (Married)		Ref.		
Single	0.378	1.46***	(1.27; 1.68)	<0.001
Widower	0.270	1.31*	(1.04; 1.65)	0.023
Legally separated	0.249	1.28*	(1.00; 1.64)	0.048
Divorced	0.429	1.54***	(1.29; 1.83)	<0.001
Study Level (University)		Ref.		
Primary	0.727	2.07***	(1.65; 2.59)	<0.001
Secondary	0.527	1.69***	(1.36; 2.10)	<0.001
Baccalaureate	0.334	1.40**	(1.11; 1.76)	0.005
Vocational Training	0.273	1.31*	(1.05; 1.65)	0.018
Constant	-5.485	0.00***		<0.001

β (Beta); OR (Odds ratio); Ref. (Reference group); CI (Confidence interval); p (p-value); * (p-value<0.05); ** (p-value<0.01); *** (p-value<0.001).
